# Data and information needs of policymakers for palliative cancer care: a multi-country qualitative study

**DOI:** 10.1186/s12911-021-01555-1

**Published:** 2021-06-15

**Authors:** Eve Namisango, Lauren Ramsey, Adlight Dandadzi, Kehinde Okunade, Bassey Ebenso, Matthew J. Allsop

**Affiliations:** 1 African Palliative Care Association, Kampala, Uganda; 2 Bradford Institute for Health Research, Bradford, UK; 3 University of Zimbabwe-Clinical Trials Research Centre ZW, Harare, Zimbabwe; 4 College of Medicine, University of Lagos, Lagos, Nigeria; 5 Nuffield Centre for International Health and Development, Leeds Institute of Health Sciences, University of Leeds, Leeds, UK; 6 Academic Unit of Palliative Care, Leeds Institute of Health Sciences, University of Leeds, Leeds, UK

**Keywords:** Cancer, Palliative care, Sub-Saharan Africa, Policymaker, Decision making

## Abstract

**Background:**

Despite regional efforts to address concerns regarding the burden of advanced cancer in Africa, urgent attention is still required. Widespread issues include late symptom presentation, inaccessibility of palliative care services, limited resources, poor data quality, disparity in data availability, and lack of stakeholder engagement. One way of helping to address these issues is by understanding and meeting the data and information needs of policymakers in palliative cancer care.

**Aims:**

To explore the views of policymakers regarding data availability, data gaps and preferred data formats to support policy and decision making for palliative cancer care in Nigeria, Uganda and Zimbabwe.

**Methods:**

A secondary analysis of interview data collected as part of a cross-sectional qualitative study that aimed to explore the data and information needs of patients, policymakers and caregivers in Nigeria, Uganda and Zimbabwe. Framework analysis, guided by the MEASURE evaluation framework, was used to qualitatively analyse the data.

**Results:**

Twenty-six policymakers were recruited. The policymakers data and information concerns are aligned to the MEASURE evaluation framework of data and information use and include; assessing and improving data use (e.g. low prioritisation of cancer); identifying and engaging the data user (e.g. data processes); improving data quality (e.g. manual data collection processes); improving data availability (e.g. the accessibility of data); identifying information needs (e.g. what is ‘need to know’?); capacity building in core competencies (e.g. skills gaps); strengthening organisational data demand and use (e.g. policy frameworks); monitoring, evaluating and communicating of data demand and use (e.g. trustworthiness of data).

**Conclusions:**

We present evidence of data sources, challenges to their access and use, guidance on data needs for policymakers, and opportunities for better engagement between data producers, brokers and users. This framework of evidence should inform the development of strategies to improve data access and use for policy and decision making to improve palliative cancer services in participating countries with relevance to the wider region.

## Background

The burden of cancer is increasing in Africa, exemplified by over 770,000 new cases and over 514,000 deaths recorded in 2018 alone [1]. A regional increase in annual incidence and cancer-associated mortality are projected to rise to 1.28 million cases and over 970,000 deaths by 2030 [2]. Regional efforts to address these concerns are intensifying, evidenced by the development and implementation of cancer control strategies [3]. Cancer care is characterised by late-stage clinical presentation, limited funding and restricted access to curative therapies with 80% of cancers on the continent advanced and incurable at the time of detection and diagnosis [4]. Palliative care—the prevention and relief of physical, emotional, social, or spiritual suffering associated with any chronic or life-threatening illness, beginning from the time of diagnosis—is a vital and fundamental component of the basic and essential services within Universal Health Coverage [5] and a realistic response to support equitable, accessible and cost‐effective interventions to relieve the suffering associated with any serious or life-threatening illness. Independent of cancer prevention and treatment efforts, the multitude of complex symptoms and concerns experienced by patients with cancer can be relieved by palliative care [6]. Palliative care should thus be integrated within cancer care and all associated policies for optimal outcomes [6, 7]. Despite this, palliative care remains underdeveloped in the region, with a lack of useful, relevant and reliable data to guide the development of appropriate and effective health services for palliative cancer care [8]. As countries gain momentum in developing palliative care and its development to support people with advanced cancer and their families, the pivotal role of health information systems should not be neglected [9].

Robust health systems rely on quality data at all levels of service delivery, for efficient and evidence-based decision making [10]. However, policymakers, planners and decision-makers in resource-limited settings often face challenges in accessing quality data to evidence and inform planning, decision making and resource allocation [11, 12]. This may result in inefficient use of resources, and pose potential risks of harm to the general population. Both a lack of data to support decision making, and the absence of a culture in which evidence-based informed decisions are embraced are significant problems in Africa [13]. Emerging specialities such as palliative cancer care face more challenges during the nascent stages of developing information systems [14]. For example, the availability of quality cancer-related data for planning and decision making in Africa remains limited [12] and the currently available registers remain localised, leading to the exclusive recording of statistics. For example, in 2012, about 60% of the countries in sub-Saharan Africa (SSA) were contributing to the cancer registry database, with minimal progress of only two additional contributors in 2014 [15]. Even where such registries are available, the data quality is poor, necessitating the use of modelling to estimate the extent of the cancer burden [15]. A means of improving knowledge and access to health information, alongside cost savings and efficiencies in health services delivery, is through the use of digital technology. Digital technology, or digital health in its broadest sense, is defined as “the use of information and communications technology in support of health and health-related fields” [16]. Development of approaches that capitalise on digital technology is a high priority for the World Health Organization who has recommended their use as an approach that can support health systems strengthening in low- and middle-income countries [16]. Increasing regional efforts to improve the quality of data in the health sector continue [17], following digital health innovations such as the district health information software (DHIS-2) which is the world's largest health management information system (HMIS) platform. This is in use by 72 low and middle-income countries [18] and is now being adopted across several African countries [18]. Furthermore, in the field of cancer, regional cancer registries are a promising initiative for increasing access to data for decision making although data quality remains a major concern compromising the extent of its use for evidence-based decision making [19]. Whilst there is an increase in the uptake and use of these data for planning, decision making and quality improvement, this could be occurring in a sub-optimal manner without meaningful stakeholder engagement [11]. Therefore, strategies that increase engagement and interaction between knowledge-producers and knowledge-users may improve production of data which meet the needs of policymakers. To address this gap in the evidence base, we sought to explore the views of policymakers regarding data availability, data gaps and preferred data formats to support policy and decision making for palliative cancer care in Nigeria, Uganda and Zimbabwe.

## Methods

### Study design

This is a secondary analysis of data collected as part of a larger multi-country, cross-sectional, qualitative study [14]. This involved interviews with stakeholders in cancer care across Nigeria, Uganda and Zimbabwe, including a total of 26 policymaker interviews, which are the key focus of this analysis. Policymakers were sampled for a district or national level role, and a focus on cancer, non-communicable diseases or digital health and technology. Participants were interviewed to help determine the optimal mechanisms through which patient-level data could be used in the development and delivery of palliative cancer care in SSA. Following initial analysis for the main study [20], a rich dataset remained that included insights into policymaker needs for data to inform decision making for palliative cancer care that had not been reported within a higher-level analysis of multiple stakeholder perspectives. A secondary analysis was conducted using a sub-sample of only policymaker data from the parent study, to address the specific aims of this study, and to re-analyse the data to bring new substantive insights and generate new knowledge on the perspectives of policymakers. While the main analysis [20] considered the rich data set as a whole, secondary data analysis enabled an in-depth understanding of the particular needs for data and information to inform policymaking and delivery of palliative care services to support patients with advanced cancer [21]. Secondary analysis of qualitative data is increasingly recognised as an effective means of adding value to original research by bringing new substantive and methodological insights, maximising learning from existing data, and informing health policy [22, 23].

### Procedure and analysis

In-depth, 1:1, face-to-face semi-structured interviews with policymakers were conducted between February and August in 2019. Interviews were audio-recorded, transcribed verbatim, translated into English where necessary (from Luganda in Uganda and Shona in Zimbabwe) and uploaded to NVivo version 12. The interviews lasted an average of 60 min. The dataset and research aim was explored against a rubric to provide assurance of its fit and relevance to this secondary analysis, the general quality of data, the trustworthiness of the original dataset and the dataset timeliness [24].

A framework analysis approach was used [25] to focus on the needs for data and information to inform decision making around the provision and delivery of palliative cancer care. Initially, transcripts were read carefully several times by three authors independently (EN, LR, MJA), to gain a holistic view and achieve immersion within the data, one of whom also conducted the interviews (EN). Data were then split between three authors according to country to make descriptive notes of initial impressions, explore commonalities and differences, and identify initial codes inductively. Regular data analysis meetings were held to review the proposed codes and make comparisons within and between the three countries. Using a deductive approach, the identified codes were mapped on to the conceptual framework for data demand and information use in the health sector [26]. The MEASURE framework highlights factors to consider in the identification of opportunities for and constraints to effective and strategic data collection, analysis, availability, and use. Framework analysis was used as its systematic approach facilitated inclusion of all members of the multi-disciplinary project team, and enabled comparison and contrasting of data by themes across many cases whilst accommodating individual perspectives and augmentation of the framework in response to the interview data [27]. To ensure all relevant information was captured, including that outside of the scope of the framework, a code labelled ‘other’ was used. This was an iterative process of consulting the transcripts, the inductive coding and the conceptual framework to ensure that decisions were grounded in the data. The findings are reported in alignment with the consolidated criteria for reporting qualitative research (COREQ) [28].

### Ethics considerations

Ethical approvals were obtained from each participating country including the Institutional Review Boards of University of Leeds (Ref: MREC 18-032), Research Council of Zimbabwe (Ref: 03507), Medical Research Council of Zimbabwe (Ref: MRCZ/A/2421), Uganda Cancer Institute (Ref: 19-2018), Uganda National Council of Science and Technology (Ref: HS325ES) and College of Medicine University of Lagos (Ref: HREC/15/04/2015). The project was aligned with the Medical Research Council good research practice guidelines [29] and H3 Africa framework for conducting ethically responsible biomedical research [30]. Participant consent included willingness for interview data to be used for secondary analyses relevant to the original research questions.

## Results

A total of 26 participants were recruited, and of these, just over half (53.85%) were females. The majority (50%) were doctors with at least 11 years’ experience at policy level. Participants were recruited from different levels of operation i.e. national ministry of health, district and in charge of cancer, non-communicable diseases or digital health technology portfolios. Study participant characteristics are outlined in Table 1.

**Table 1:** Characteristics of study participants

Characteristic	N (%)
*Sex*	
Male	12 (46.15%)
Female	14 (53.85%)
*Professional background*	
Doctor	13 (50.00%)
Nurse	3 (11.54%)
Pharmacist	3 (11.54%)
Public Health Specialist	2 (7.69%)
Health Informatics	2 (7.69%)
Statistician	1 (3.85%)
Surveillance Manager	1 (3.85%)
Social worker	1 (3.85%)
*Country*	
Nigeria	5 (19.23%)
Uganda	11 (42.31%)
Zimbabwe	10 (38.46%)
*Level of operation*	
National	15 (57.69%)
District	2 (7.8%)
In charge of cancer, non-communicable diseases or digital health and technology	9 (34.51%)

The findings are reported according to the conceptual framework for data demand and information use in the health sector. Within each of the framework concepts, we describe the thematic and sub-thematic areas in detail.

### Assessing and improving data use context

This theme relates to organisational, technical and behavioural factors that affect decision making, for example, characteristics and dynamics within the organisation that influence decision making. Within this theme, respondents raised concerns around working in silos, which makes data sharing for decision making difficult to achieve. This may be due to the vertical programming, but also the weak partnership between the private and non-private actors in cancer care.

*“Challenges will always be there. Private institutions are not providing us with adequate support in terms of reporting so, what we have as data is, you know, the Non-Governmental Organisations that are not for profit, it’s private not for profit.” respondent* 02-Zimbabwe

*“So, you find HIV implementing partners do not want to bring hepatitis on board, yet these are diseases of the same model in transmission. We use close to the same drugs monitoring so you find that integration is a way to go but there is resistance, of course from implementing partners because they don’t have these in their strategic plan probably, so we are trying to get people or partners on board and say look here we need to integrate there is need for integration, because this is one patient we are looking at and they are so many others.” respondent 01-Uganda*

In addition, shortages of funding for palliative cancer care was a barrier. The countries equally have competing priorities in the health sector, all of which require funding. Given that cancer care is relatively expensive, governments find it challenging to provide reasonable budgets to meet the increasing demand for care. As such, cancer care is largely self-funded by patients, and resultantly, a limited pool of money is available to support service development.

*“…[in] Lagos state we have 24 million people who we look after and we have to touch on a lot of emergency diseases, we’ve just managed to handle monkey pox, and outbreak of malaria in the last one month and we’ve curtailed [them] successfully, a lot of times the populace don’t even know the challenges we go through and all the battles we have trying to curb all [these] things to the point that the outbreaks become non-existence.” respondent* 03--Nigeria

*“The critical problem is that patients pay from pocket… no matter the information we have, we won’t do too much if patients keep paying from pocket… I think it is very important that we improve on our health insurance scheme.”* Respondent 03- Nigeria

Low prioritization and an absence of awareness about the importance of data for palliative cancer services was prevalent in Zimbabwe and Nigeria. The need to demonstrate the difference palliative care makes was also emphasised, as this is commonly required to justify resource allocation.

*“For us it’s a big learning curve as a country because despite being home to the oldest hospice on the African continent we still largely perceive palliative care as a service for the elite, and then not for children so it’s something that we really have to spread awareness but also demonstrate the difference that palliative care makes to both children and adults.”* Respondent 02- Zimbabwe

### Identifying and engaging data users and data producers

This theme is concerned with who the data-users and producers are, the processes around data creation and sharing at different levels of an organisation, which data is available and whether data needs are being met.

To map the data producers, we focused on the data sources and data flow. In the three countries, there was a clear hierarchy for data flow, from the source of data generation (within the district), which is commonly at the service delivery level or cancer registry centres, to the national level. These data are gathered in disparate ways and are largely in paper-based formats. Across the three countries, respondents were able to identify and demonstrated a clear understanding of potential data sources.

*“…so how do I get data, it can be through support supervision when I go to the site, [health facility] and I get the data through a paper based [format], .. if I want to know maybe in Kibuko health centre IV, how many patients have been enrolled for hepatitis B treatment, that could actually click or go to the DHIS 2 and find out.., by the district how many people are actually are positive and so I could get it electronically through DHIS 2 or through my support supervision where I go and use the paper based.”* Respondent 01-- Uganda

Although surveys and cancer registers were the most dominant sources of routine data, other data sources include; facility registers, cancer care routine statistics (e.g. hospital registers), supervision reports, district health information system reports, community surveillance reports, reports from other stakeholders (e.g. HIV care providers and World Health Organization) and national surveys (e.g. the census and demographic household surveys). Some participants expressed preferences for national surveys, which they premise are collected over time and by credible institutions, hence appropriate for decision making.

*“We have a monthly activity data report that we get from all the facilities and all the local governments in the state. Now the facility data report we get from the err state facility, the local government that one covers all even the public and the private facilities in the state, so we have that every month. Now we also have NHMIS (national health management information system) monthly report that we get, that one is of course on digital platform.”* Respondent 03- Nigeria

*“…currently the information they are using is partially digital, but most of it is really manual. Yeah, and most of it is based on surveys which come…is it within every 2 years? I think every 2years, the national health survey of DHIS-2.* …*Secondly, they also trust universities, there are people professors and so on and other academics who have done credible work and there’s track record about them and they know about it. And they also, you know, dig behind the data produced by these people. It’s not just … they don’t just come say, okay I have discovered something.”* Respondent 01-Uganda

With regard to engaging data-producers and users, we noted emerging initiatives for merging data sources, refining or scaling up data sources to allow for increased access to data for decision making and encouraging all service providers to collect data. For example, Uganda is developing an atlas to show the type of digital applications available and their use, given the increased growth in digital platforms and approaches. In Nigeria, a law for research was introduced, which requires every local government in the state to provide services for palliative care and must collect routine statistics. Such statistics include age, sex, marital status, state of origin, residential address, the parity (if female), occupation, and family history. Respondents also noted an increase in the uptake of generating evidence locally.

*“…we started working on the digital health atlas, a tool that should enable leaders within the ministry of health, like the PS [Permanent Secretary] and other health managers, to kind of try to see what digital health tools are in the country… what do they do? What problem are they solving? It’s a tool that we started off and left for the team there, to you know, progress with it.”* Respondent 01 -Uganda

*“…research centre law, there is a law in the state now, and this is part of the provisions in the law that every local government in the state must provide services for palliative care.”* Respondent 03-Nigeria

### Data quality

This theme includes completeness, accuracy and timeliness of data. Data quality was a cross-cutting theme across the three countries. Participants raised concerns around data incompleteness, which was attributed to a broad range of factors including poor documentation practices, lacking knowledge regarding the national indicators and a lack of core competencies in collecting the associated data.

*“That is what is expected however… you may actually find that some of the health facility officers in health facilities have no clue whether those indicators actually are in the HMIS forms, and many times they don’t report on it. So, we find challenges to report on it, but for those who have reported on it we use those figures to inform policies.” 01- Uganda*

In addition, respondents also noted the long manual processes, which make it difficult for care providers to consistently keep track of the patients’ medical history as another contributing factor to data incompleteness. More so, the data are largely without subsequent area-level aggregation, leading to under-reporting of epidemiological data.

*“We should be able to say on an annual basis… how many of the different cancers did we diagnose in Marondera [province]… that would then be able to tell us what sort of interventions we need to be putting in place. Right now, we may not be able to do that. So, I am saying we still have a challenge there because most of it is paper based, it’s not disaggregated and that limits the extent to which we can make decisions.” 02- Zimbabwe*

Respondents also raised concerns about data accuracy and timeliness. For example, there is an over reliance on WHO estimates, which are based on morbidity data and statistical modelling. The widespread paucity of cancer registers across countries to capture data on incidence and poor practices in recording cancer-related mortality all lead to underestimates in reporting of incidence and prevalence. Data are also captured manually and are required to go through a series of processes before they can be accessed for timely use. For example, the hard copies were expected to be taken to specified centres for data entry, then later collated at higher level centres, where data cleaning may happen, after which the data can be accessed via an online system. This usually causes a considerable time lag, and as such, data cannot be used for timely planning and decision making.

### Improve data availability

This theme assesses the extent to which the data that is received is synthesised, communicated in a way that is accessible to individuals at different levels of the organisation and whether the data meets the information needs at different levels of the organization.

Participants raised a concern around data availability. Firstly, cancer-related data are not available in appropriate formats and at times the desired data are not what is available. For example, there are some decisions which require unavailable longitudinal data. Additionally, available data is often in paper formats, posing challenges for data mobility and ease of access.

*“…unfortunately I don’t have information on the types of people that have these kinds of cancers, If I have report on ten cancers of the cervix, I don’t know the age group of the women that have these, I don’t know anything about their socio-economic factors, I don’t know anything about the staging of the cancers, I don’t about how it was diagnosed, I don’t know anything about the kind of treatment they’ve had, I don’t have anything. I don’t even have anything about their geographical distribution… all I have was that I have ten cases of cancer of the cervix and this all that is it.” Respondent -03- Nigeria*

The theme on variation in data needs by the level of service delivery was more pronounced in Uganda, as participants noted that patient-level data is required by middle-level managers who are directly responsible for delivering patient care and not by policymakers. They also posited that failing to mainstream data needs by the level of service delivery may lead to data bombardment which comes with time wasted in trying to identify what is relevant for their respective roles.

“*There is top management, middle management and operational at operational level because we need to address this challenge of the pain, who can address that? It is these operational managers, the front-line health workers? So for us, what we want is how do you manage this, what do you need? What do you need to manage that severity of the pain?” Respondent 01--Uganda*

### Identifying information needs

This theme assesses gaps in the information needs.

Participants noted various data-related information needs spanning the six WHO health system building blocks for health service delivery, health workforce, health information, essential medicines, health financing and leadership and governance [9]. For example, a lack of accurate morbidity data compromises policymakers’ ability to estimate the need for supplies such as opioids for pain where estimation requires robust morbidity and mortality data. The unavailability of accurate data further affects the precision of estimates for other cancer medicines and supplies.

Other data and information needs included data regarding human resource inventory, which is pivotal to effective referrals and in improving access to services by patients. The absence of data regarding the cost of cancer care was highlighted as a concern as participants felt it is difficult to implement policies which cannot be costed. Similar difficulties were expressed in trying to justify reasonable budgets for cancer care with insufficient data to demonstrate need. Different data needs for mid-level managers and commissioners included that they wanted more specific data which is not currently available to suit their specific needs.

### Building capacity in data use core competencies

This theme addresses skill availability and/or gaps among stakeholders.

Participants highlighted a stark shortfall in human resource and capacity for data collection. Some of the existing staff have not been trained in data collection and the meaning of the various indicators collected in the health information management systems, which results in poor quality data. Work pressures are also a concern, because of the expectations on health workers to care for patients and also meet the reporting and routine data collection requirements. In addition, the staff lack skills in data management and data analysis which limits their ability to use data for decision making even at the production level.

Alternative digital tests are thus proposed, to reduce the time required for data collection, and processing. Specialities such as HIV and malaria care are good examples from which the cancer speciality can learn. The HIV and malaria programmes were already using dashboards for real-time data capture and sharing.

### Strengthening organisations data demand and use infrastructure

This theme assesses the processes and systems that support or hinder the use of data in decision making e.g. policy context around eHealth.

Variations in the infrastructure available to support the strengthening of data demand and information use were apparent. For example, Uganda has an enabling policy framework with an eHealth policy and a cancer control policy which is underway, and a health sector strategic plan which is pro-data demand and information use, including the use of technology in data management, access and use. Zimbabwe and Nigeria lack formal standardised policies to support their work, although they have visibility of eHealth strategy or emerging drafts and supporting documents which are promising.

### Monitoring, evaluation and communication results of data demand and use

This theme assesses to what extent individuals within the organisation value the data including perceptions of trustworthiness, robustness, and reliability of data.

The findings regarding monitoring, evaluation and communication results of data demand and use were mixed. While data-driven decision making was valued across the three countries, there are also concerns around the trustworthiness of the data, and integrity of the data generated. There was also awareness that gaps in data quality and availability for decision making influenced trustworthiness, and indicated that more can be done.

## Discussion

With the increasing burden of cancer and predominant presentation of patients with advanced disease, the need to use data for policy and resource allocation cannot be under-estimated [31]. Given that most patients present late with limited curative options, integrated palliative cancer care must be prioritised for optimal pain and symptom management. This study provides robust evidence regarding the available data, data needs and priorities for scaling up data availability and its use by policymakers for decision making as they embrace the integrated model of care. The challenges identified are not unique to cancer care, and there are opportunities to learn from HIV care. We found concerns around organisations working in silos and subsequently hampering the feasibility of data sharing across partner organisations. This can be possibly explained by the vertical programming approach which has been commonly used by funding agencies in African settings. The continued move towards integrating palliative care into cancer care services and collaborative initiatives to get cancer and palliative care-related indicators into the national health information systems are promising practices which should be strengthened to address this challenge. Working in silos could also be addressed by promoting deliberate efforts for various stakeholders (including funding agencies) to share their data in a transparent manner [13].

The lack of political will to fund palliative cancer care has also been identified as a systemic problem and a barrier to cancer care development [32]. With limited funding, patients and their families continue to endure catastrophic financial expenditure in accessing care and treatment [33]. Countries like Rwanda prioritising basic facilities needed for cancer care could be good learning examples [32]. Increasing access to quality data highlighting the magnitude of the problem and plight of catastrophic expenditure could also help inform advocacy efforts towards increased public financing for cancer care [33]. This is especially important as low and middle-income countries adopt Universal Health Coverage, and the evidence for an investment case for palliative cancer care is a pressing priority to inform regional advocacy efforts.

Our findings suggest the presence of a clear data flow structure from the production centres to the national level. The pathway for mapping the actors in the generation and use of data is thus easier to achieve. Given that the meaningful engagement between the partners who generate knowledge and those who use it is central to the uptake of data and information used in planning and decision making, platforms for interaction should be availed from the point of planning for the data collection. This should occur throughout the stages of data collection, data cleaning, synthesis, analysis, and interpretation to allow for its effective use for policy, planning and decision making [34]. Leaving data producers out of the decision-making processes is common practice yet undesirable as it may result in policies that are not practical or those that have limited practical relevance for those meant to implement them. As such, a two-way communication should be adopted as best practice [35, 36]. These interactions should be prioritised for funding as the data has an impact on service delivery although this is at times not recognised.

Another important finding relates to data quality issues, and this spans across the thematic areas of completeness, accuracy and timeliness. To begin with, data completeness should be understood from two perspectives i.e. regional coverage in-country and completeness in terms of the data fields for the data which is collected. The concern that cancer registers are localised leads to under-reporting of epidemiological data, as such the prevalence and incidence of cancer cannot be accurately recorded [15]. This may be explained by the localised approach to cancer care, which is biased to urban settings. This is a trend that should be reversed, as accurate data is needed to determine the burden of cancer and resultantly plan for the services. As countries decentralise cancer care for equitable access, registration should be expanded to support registration of new cases and other epidemiological details. For data completeness, the interventions of training on best practices in data collection, meaningful feedback for relevant staff and regular data reviews and audits can be  effective at the level of data production i.e. health facilities [37].

The second data quality concern was accuracy, a serious threat to the use of data in decision making. For example in a survey that aimed to appraise the quality of African cancer registration systems, 19 of the 26 registers failed to meet more than five of the 15 quality criteria, which included accuracy, timeliness, validity and comparability: “a measure of how well data classification and coding conforms to international guidelines and best practice” [19]. Existing literature also links poor data accuracy to poor training of the people that collect the data and at times surrounding pressures at work. Interventions such as regular data quality audits and regular data review meetings have proved useful for improving data quality in Africa [38]. The latter is especially common where staff must care for patients and also address routine reporting data requirements. This concern is not unique to cancer, and indeed this growing speciality can learn a lot from the HIV field, where several interventions have been honed to improve data accuracy [38]. Lastly, timeliness of data was also a key finding of this study, because the data management processes are manual, a finding which is in line with existing evidence [15]. Poor performance on the timeliness of data leads to major delays from the time of data generation to availability for use in planning and decision making. Embracing digital strategies that allow for the use of digital technology in data collection to allow for real time access to data for planning and decision making should be further prioritised to bridge this gap [34].

In regard to data availability, largely, respondents appreciated the increased availability of data, most especially the epidemiological data, although data quality concerns still remain a challenge. There is a need for patient-level data, data on costing and human resource inventory for cancer care, and palliative cancer care specifically. These data are critical to decision making and planning. For example, to advance regional advocacy for Universal Health Care Coverage, service delivery and the costing of data are needed yet this remains a big gap in the sector [39]. Regarding the mapping of service delivery points as well as undertaking human resource inventories, the region should prioritise these as services are localised and identifying appropriate care centres would reduce the delays in enrolment into care and hence optimise outcomes [40]. The International Association for Hospices and Palliative Care hosts a directory of palliative care services across Africa, and this could be an example that African countries can scale up locally paired to robust geographical information technology [41].

To our knowledge this is the first study to explore policymaker preferences for data to inform palliative cancer care development in Africa. Our purposive sampling frame was achieved in the parent study, ensuring perspectives from participants of differing policymaker roles, using broad, open-ended questions about the experiences and needs of participants. Secondary analysis was conducted across a multi-disciplinary team, including interviewers (EN; AD) and research coordinators (KO, MJA) from the parent study. Limitations of the study include that the parent study had a different primary aim, meaning further elaboration of important themes and refinement of topic guides relating to the study aim could not occur.

For an effective response to data and information needs in palliative cancer care, it is important to strengthen organisation infrastructure by developing policy frameworks which support data demand and information use. Without policy frameworks, accountability can be compromised, and it is difficult to negotiate for budget allocations. Some countries have empowering frameworks in place promoting a positive organisational culture which values data and information in decision making. For example, Uganda has an eHealth policy (ePolicy) which promotes the use of technology to improve health service delivery [42]. Such initiatives should be supported in cancer care across the region, with reasonable budget allocations to support the roll-out of these policies. The findings of this study highlight the priorities for increasing access to data and its uptake for decision making in cancer care and control.

## Conclusion

From three countries in SSA, we present evidence of data sources, challenges to their access and use, guidance on data needs for policymakers, and opportunities for better engagement between data producers, brokers and users. In the SSA region, the continuing rise in cases and deaths from cancer is occurring alongside increasing engagement with digital health approaches, presenting an opportunity to enable data access and use for policy and decision making. Our data provides an initial framework to enhance evidence-informed decision making to guide the development and critical expansion of palliative care to ensure contextually-appropriate care for people living with advanced cancer and their caregivers in SSA.

## Data Availability

The datasets analysed during the current study are not publicly available due to ethical restrictions not allowing the publication of raw data in terms of interview transcripts but are available from the corresponding author on reasonable request.

## Abbreviations

COREQ: Consolidated criteria for reporting qualitative research
DHIS-2: District health information software
HMIS: Health management information system
NHMIS: National health management information system
SSA: Sub-Saharan Africa
